# Reliability and Construct Validity of the Short Physical Performance Battery in Croatian Older Adults

**DOI:** 10.3390/geriatrics11020033

**Published:** 2026-03-19

**Authors:** Tatjana Njegovan Zvonarević, Ivan Jurak, Mirjana Telebuh, Ana Mojsović Ćuić, Edina Pulić, Ivna Kocijan, Želimir Bertić, Miljenko Franić, Igor Filipčić, Vlatko Brezac, Klara Turković, Lana Feher Turković

**Affiliations:** 1 Department of Occupational Therapy, University of Applied Health Sciences, Mlinarska Street 38, 10000 Zagreb, Croatia; tatjana.njegovan-zvonarevic@zvu.hr (T.N.Z.); edina.pulic@zvu.hr (E.P.); 2 Department of Physiotherapy, University of Applied Health Sciences, Mlinarska Street 38, 10000 Zagreb, Croatia; ivan.jurak@zvu.hr (I.J.); mirjana.telebuh@zvu.hr (M.T.); 3 Department of Physiotherapy, Alma Mater Europaea University, Slovenska Street 17, 2000 Maribor, Slovenia; 4 Faculty of Health Sciences, University of Primorska, Polje 42, 6310 Izola, Slovenia; 5 Department of Biology and Physics, University of Applied Health Sciences, Mlinarska Street 38, 10000 Zagreb, Croatia; ana.mojsovic-cuic@zvu.hr; 6 Department of Anatomy and Physiology, University of Applied Health Sciences, Mlinarska Street 38, 10000 Zagreb, Croatia; ivna.kocijan@zvu.hr; 7 Institute of Public Health of Bjelovar-Bilogora County, Division of Public Health, Matice Hrvatske 15, 43000 Bjelovar, Croatia; bertic.z@gmail.com; 8 Department of Nursing, Faculty of Health Studies, University of Rijeka, Viktora Cara Emina 5, 51000 Rijeka, Croatia; 9 Department of Clinical Medicine, University of Applied Health Sciences, Mlinarska Street 38, 10000 Zagreb, Croatia; mfranic@kbd.hr; 10 Department of Orthopedics and Traumatology, Dubrava University Hospital, Avenija Gojka Šuška 6, 10000 Zagreb, Croatia; 11 Department of Orthopaedic Surgery, School of Medicine, University of Zagreb, Šalata 7, 10000 Zagreb, Croatia; 12 Institute for Integrative Medicine, Faculty of Dental Medicine and Health, Josip Juraj Strossmayer University of Osijek, Crkvena 21, 31000 Osijek, Croatia; 13 Department of Psychiatry, University of Applied Health Sciences, Mlinarska Street 38, 10000 Zagreb, Croatia; igor.filipcic@zvu.hr; 14 School of Medicine, University of Zagreb, Šalata 3, 10000 Zagreb, Croatia; 15 University Psychiatric Hospital “Sveti Ivan”, Jankomir 11, 10090 Zagreb, Croatia; 16 Department of Social Gerontology, Alma Mater Europaea University, Slovenska Street 17, 2000 Maribor, Slovenia; brezac.vlatko@gmail.com; 17 Health Care Institution ‘Medici’, 10000 Zagreb, Croatia; kt.turkovic@gmail.com; 18 Department of Chemistry, Biochemistry and Clinical Chemistry, University of Applied Health Sciences, Mlinarska Street 38, 10000 Zagreb, Croatia

**Keywords:** short physical performance battery, SPPB, physical performance, older adults, reliability, validity, Croatia

## Abstract

**Background**: Population aging represents a major public health challenge, accompanied by an increasing prevalence of chronic diseases and age-related functional decline. Declines in lower-extremity physical function are particularly important, as they are strongly associated with mobility limitations, loss of independence, increased risk of falls, hospitalization, and mortality in older adults. Reliable and valid tools to assess physical performance are therefore essential in both clinical and research settings. The Short Physical Performance Battery (SPPB) is a widely used instrument for assessing lower-extremity physical performance in older adults and is recommended within the diagnostic algorithm of the European Working Group on Sarcopenia in Older People (EWGSOP2) for evaluating physical performance severity. However, the SPPB has not yet been psychometrically validated in the Croatian older population. This study aimed to evaluate the reliability and validity of the SPPB in Croatian older adults. **Methods**: This study examined the metric properties of the SPPB in a sample of 153 older adults recruited from nursing homes and community settings. **Results**: The SPPB demonstrated acceptable internal consistency (Cronbach’s alpha = 0.74) and good test–retest reliability (ICC = 0.893) for the total score. Convergent and construct validity were supported by significant associations with established measures of functional mobility and muscle strength. **Conclusions**: The Croatian version of the SPPB is a reliable and valid instrument for assessing lower-extremity physical performance in older adults. Its use is supported in clinical practice and research settings in Croatia. Further studies should examine responsiveness and predictive validity in nationally representative samples.

## 1. Introduction

Population aging represents one of the major public health challenges of contemporary societies, accompanied by an increased prevalence of chronic diseases and age-related conditions. Among these, age-related declines in muscle strength, mobility, and overall physical performance contribute substantially to functional decline, frailty, falls, loss of independence, and increased mortality in older adults [1,2]. Muscle strength and physical performance are recognized as key determinants of health outcomes in later life [2,3]. Consequently, the assessment of physical performance has become an essential component of geriatric evaluation, as it reflects the integrated function of multiple physiological systems involved in mobility, balance, and functional independence [3,4].

Physical performance refers to objectively measurable whole-body functioning related to mobility and lower-extremity function. Impairments in physical performance are strongly associated with adverse outcomes in older adults, including falls, disability, institutionalization, reduced quality of life, and mortality [5,6,7,8]. Standardized performance-based tests enable reliable and objective assessment of these impairments and support early identification of individuals at risk of functional decline [9,10].

SPPB is one of the most widely used instruments for assessing lower-extremity physical performance in older adults. It consists of three components, namely standing balance, gait speed, and repeated chair stands, providing a composite measure of lower-limb function. Numerous international studies have demonstrated reliability and validity across diverse populations and settings, and it has been shown to predict functional decline, disability, hospitalization, and mortality [8,11,12,13,14,15,16,17,18,19]. As a result, the SPPB is recommended by EWGSOP2 as a measure of physical performance for evaluating sarcopenia severity [3].

Despite its widespread use, the meaningful application of the SPPB in both research and clinical practice requires validation within specific linguistic, cultural, and healthcare contexts. Measurement instruments must demonstrate adequate reliability and validity to ensure accurate interpretation of results and to support evidence-based decision-making [20,21,22,23,24]. In geriatric research, where even small changes in functional status may have substantial clinical consequences, the use of validated tools is particularly important [25].

To date, no standardized validation of the SPPB has been conducted in the Croatian older adult population. Establishing its metric properties is essential to enable reliable assessment of lower-extremity physical performance and to support sarcopenia-related research and clinical evaluation in Croatia. For this purpose, convergent and construct validity were examined using established functional measures, including handgrip strength assessed by dynamometry and the Timed Up and Go (TUG) test, which have previously demonstrated significant associations with physical performance and sarcopenia-related outcomes [3,26,27,28,29,30]. The aim of this study was to evaluate the reliability and validity of the Croatian version of the SPPB in older adults. Although the SPPB is recommended within the EWGSOP2 algorithm as a measure of physical performance severity, the present study focused exclusively on its psychometric properties.

## 2. Materials and Methods

### 2.1. Participants

A total of 153 participants (32 males and 121 females), with a mean age of 81.4 years (SD = 7.69), were included in the study. This sample size is generally considered adequate for psychometric validation of short scales such as the 3-item SPPB. All participants provided written informed consent prior to participation. The study included 60 participants from the retirement home “Elderly Care Home Centar” (City of Zagreb, Croatia), 22 from “Elderly Care Home Park” (City of Zagreb, Croatia), 43 from “Elderly Care Home Bjelovar” (Bjelovar–Bilogora County), and 28 community-dwelling older adults from Bjelovar (Bjelovar–Bilogora County). Participants were recruited using convenience sampling through collaboration with selected nursing homes and community organizations. Therefore, the sample may not be fully representative of the broader older Croatian population. Participants aged 65 years and older who were able to stand and walk independently, understand verbal instructions, and provide written informed consent were eligible for inclusion. The study was conducted between March and September 2025. Exclusion criteria included acute illness; severe cardiovascular, respiratory, orthopedic, neurological, or psychiatric conditions that could interfere with study assessments; and being bedridden or immobile, having cognitive impairment, or any condition limiting the ability to understand and follow instructions. Three participants who did not meet the inclusion criteria were excluded. The test battery was administered to both institutionalized and community-dwelling older adults from the City of Zagreb, Zagreb County, and Bjelovar–Bilogora County. Data were collected to assess functional status and to examine the metric properties of the SPPB in the local context. The sample size was based on participant availability through collaboration with local centers for older adults.

Inclusion criteria: age ≥ 65 years; ability to stand and walk independently; ability to understand verbal instructions; written informed consent. Exclusion criteria: acute illness; severe cardiovascular, respiratory, neurological, orthopedic, or psychiatric conditions; cognitive impairment; immobility or inability to follow instructions. These criteria ensured that all participants could safely and reliably complete the SPPB and associated functional tests.

### 2.2. Translation and Adaptation Procedure

The translation and cultural adaptation of the Short Physical Performance Battery (SPPB) were conducted following internationally accepted guidelines for the cross-cultural adaptation of health-related measurement instruments, applied to the translation of test instructions and administration procedures [31]. Permission from the copyright holders was obtained prior to initiating the process. A dual independent forward translation of the original English version into Croatian was performed by one healthcare professional and one professional translator without a medical background. The two translations were compared and reconciled into a single synthesized version to ensure conceptual equivalence. Subsequently, two independent translators, blinded to the original version, conducted a back-translation into English to verify consistency with the source version. The prefinal Croatian version was reviewed by a multidisciplinary expert committee to evaluate the semantic, idiomatic, conceptual, and cultural equivalence of the translated instructions and procedures. The expert committee consisted of seven members, including a physiotherapist, an occupational therapist, a psychiatrist, a gerontologist, an orthopedic surgeon, and a research methodologist. Members were selected based on their experience in geriatric care, familiarity with functional assessment instruments, and expertise in health research methodology. As the SPPB assesses basic lower-extremity functional tasks that are routinely performed in daily life, no substantial cultural adaptations were required. Comprehensibility and feasibility were further evaluated through pilot testing of the pre-final Croatian version of the SPPB on 15 participants, which showed that the instructions were clear and understandable and the tasks could be performed without difficulty. Following completion of the translation and adaptation process, psychometric validation of the Croatian version of the SPPB was conducted, including the assessment of reliability and validity in accordance with established methodological criteria [8].

### 2.3. Data Collection

Ethical approval for the study was obtained from the Ethics Committee of the University of Applied Health Sciences, Zagreb, Croatia (29 December 2023; CLASS: 602–03/23–18/933; REG. NO.: 251–379–10–23/02), the Ethics Committee of the City of Zagreb (11 March 2025; CLASS: 550–01/25–001/130; REG. NO.: 251–09–12–2/003–25–4), and the Administrative Board of the Bjelovar Pensioners’ Association (decisions dated 3 March 2025). All participants voluntarily agreed to participate in the study and provided written informed consent. All data were collected and processed anonymously. During data collection, participants were informed about the purpose and procedures of the study. Functional assessments were subsequently performed, including balance testing, gait assessment, repeated chair stand performance, the Timed Up and Go (TUG) test, and handgrip strength measurement using a dynamometer. All assessments were conducted by trained professionals following standardized protocols. After testing, participants received basic feedback on their functional performance and muscle strength, and general recommendations regarding the importance of physical activity and maintenance of muscle function were provided when appropriate. For the assessment of test–retest reliability, a subset of 31 participants was selected from the total sample using stratified random sampling to ensure representation across different levels of functional capacity and muscle strength. The SPPB was re-administered after a 14-day interval, which is consistent with methodological recommendations aimed at minimizing recall bias while maintaining clinical stability of the measured construct [14]. No interventions that could affect functional capacity or muscle strength were conducted during the interval between testing and retesting, thereby minimizing the risk of changes in participants’ functional performance. The same trained assessors conducted both the initial assessment and the retest, following identical standardized protocols. Evaluators were blinded to the participants’ previous test results during the test–retest assessments to minimize bias. All study procedures were conducted in accordance with the ethical principles of the Declaration of Helsinki [32].

### 2.4. Instruments

#### 2.4.1. Short Physical Performance Battery (SPPB)

The Short Physical Performance Battery (SPPB) is a standardized performance-based test used to assess lower-extremity function in older adults. It consists of three components: a balance assessment, a 4 m gait speed test, and a five-time sit-to-stand test. Gait speed and chair stand performance were measured in seconds, while balance performance was scored according to the SPPB protocol (0–4 points). The total SPPB score ranges from 0 to 12 points, with higher scores indicating better physical performance. An SPPB score ≤ 8 points indicates reduced physical function, while a gait speed ≤ 0.8 m/s reflects reduced functional capacity [3,8].

- Balance Test

Static balance was assessed using three standardized standing positions: side-by-side, semi-tandem, and full tandem stance. Each position was held for up to 10 s following the SPPB protocol. The test was terminated if the participant moved their feet, stepped, or required external support. Balance performance was scored from 0 to 4 points according to the SPPB scoring criteria, with higher scores indicating better postural stability [8]. All assessments were conducted by trained physiotherapists or occupational therapists following standardized safety procedures.

- Four-Meter Walk Test

Gait speed was assessed using the 4 m walk test as part of the SPPB. Participants were instructed to walk at their usual pace along a clearly marked 4 m path. Timing began when the first foot crossed the starting line and ended when the first foot crossed the finish line. Two trials were performed, and the faster trial was used for scoring. Gait speed was calculated in meters per second (m/s) and scored from 0 to 4 points according to the SPPB protocol [8].

- Sit-to-stand Test (STS Test)

Lower-extremity strength and functional performance were assessed using the five-time sit-to-stand test included in the SPPB. Participants were seated in a standard chair with their arms folded across the chest and instructed to stand up and sit down five times as quickly as possible without using their arms. Time was recorded in seconds and scored according to the SPPB protocol (0–4 points), with shorter completion times corresponding to higher scores. A completion time greater than 15 s indicates low physical performance according to EWGSOP2 criteria [3,8].

#### 2.4.2. Measurement of Handgrip Strength Using a Dynamometer

Handgrip strength (HGS) was measured using a calibrated digital hand dynamometer (Saehan DHD-1, Glanford Electronics Ltd., Scunthorpe, UK). Measurements were performed on the dominant hand, with participants instructed to exert maximal force. Three trials were conducted, and the mean value was used for analysis. Low muscle strength was defined according to EWGSOP2 cut-off values (<27 kg for men and <16 kg for women) [3].

#### 2.4.3. Timed Up and Go Test (TUG)

Functional mobility was assessed using the Timed Up and Go (TUG) test. Participants were instructed to stand up from a standard chair, walk three meters at their usual pace, turn, return, and sit down. The total time to complete the task was recorded in seconds [28].

The TUG test and handgrip strength were selected as convergent measures because they are standardized, widely validated indicators of functional mobility and muscle strength in older adults and are conceptually related to lower-extremity performance assessed by the SPPB [9].

### 2.5. Statistical Analysis

Descriptive statistics were used to summarize demographic, anthropometric, and functional data, and results are reported as means and standard deviations (SDs), medians with minimum and maximum values, or frequencies and percentages, as appropriate. Internal consistency of the SPPB was evaluated using Cronbach’s alpha and item–total correlations. Cronbach’s alpha for deleted items and split-half reliability (including Guttman’s λ coefficients) were also computed. Cronbach’s alpha was interpreted according to conventional thresholds (≥0.90 excellent; 0.80–0.89 good; 0.70–0.79 acceptable; 0.60–0.69 questionable; <0.60 poor). The average inter-item correlation was additionally reported as an indicator of item homogeneity. Convergent validity was assessed by correlating SPPB total and subtest scores with the Timed Up and Go (TUG) test, using Spearman’s rank correlation coefficients (ρ). Test–retest reliability was examined in a subsample of 31 participants. Intraclass correlation coefficients (ICCs) were calculated using a two-way random-effects model (absolute agreement, single measures; ICC (A,1)) for the total SPPB score and each subtest. Participants were asked at the time of retesting whether any acute illness, injury, hospitalization, or significant change in health status had occurred during the 14-day interval. No such events were reported. Agreement between test and retest scores was further evaluated with Bland–Altman analysis, calculating mean differences and 95% limits of agreement. Construct validity was explored using factor analytic methods. Sampling adequacy was examined with the Kaiser–Meyer–Olkin (KMO) test and Bartlett’s test of sphericity. Exploratory factor analysis (EFA) with principal axis factoring and oblimin rotation was performed to evaluate the latent structure of the three SPPB subtests. All statistical tests were two-tailed, and the threshold for significance was set at α = 0.05. All analyses were performed in R (version 4.3.1; R Core Team, Vienna, Austria).

## 3. Results

### 3.1. Descriptives

Table 1 summarizes the basic characteristics of the study sample (*n* = 153). Most participants were women (79.1%), with men comprising 20.9%. The mean age was 81.4 years (SD 7.69; range 65–97). Average anthropometric values were 165 cm in height (SD 8.06), 75.6 kg in weight (SD 13.0), and 27.8 in body mass index (SD 4.62). Dominant handgrip strength averaged 21.4 kg (SD 6.95; range 3.2–45.2), with 22.2% of participants classified as weak according to established cut-offs.

Table 2 presents descriptive data for the SPPB and TUG tests. The mean subtest scores were 2.24 (SD 1.55) for balance, 2.64 (SD 1.07) for gait, and 1.67 (SD 1.36) for chair stand. The average overall SPPB score was 6.62 (SD 3.34; range 1–12). Categorically, 19.6% of the participants were classified as having very low function, 30.7% as low, 22.9% as moderate, and 26.8% as high functional status. Timed Up and Go performance averaged 16.0 s (SD 9.15; range 5.6–52.0). Based on established cut-offs, 27.5% of the participants were classified as having normal mobility, 52.3% as good, 11.8% as moderate, and 8.5% as reduced mobility.

### 3.2. Reliability

Table 3 summarizes internal consistency indices for the SPPB. Cronbach’s alpha was 0.74 (standardized 0.75), indicating acceptable reliability (0.70–0.79) for a short, 3-item scale. Item–total correlations ranged from 0.56 to 0.59, confirming that all three subtests contributed meaningfully to the total score. Removal of any item reduced alpha (varying from 0.63 to 0.67), supporting the retention of all items. Split-half reliability indices were high, with an average coefficient of 0.89 and Guttman’s λ values ranging from 0.65 to 0.75, consistent with good internal consistency. However, because the scale includes only three items, split-half estimates were based on unbalanced (2 vs. 1 item) splits and, therefore, likely overestimated the true reliability. In this context, Cronbach’s alpha provides a more realistic and conservative indicator of internal consistency; however, for posterity reasons, we also include the results of the split-half reliability. The average inter-item correlation was 0.50 (median 0.49), within the recommended range (0.20–0.50) for homogeneity without redundancy.

### 3.3. Convergent Validity

Table 4 presents Spearman correlations between the SPPB, its subtests, and the Timed Up and Go (TUG) test. The total SPPB score showed a moderate-to-strong negative correlation with TUG (ρ = −0.684, *p* < 0.001), indicating that higher lower extremity function was associated with faster mobility performance. Among subtests, Gait demonstrated a strong correlation with TUG (ρ = −0.752), consistent with the shared emphasis on walking speed. Chair showed a moderate correlation (ρ = −0.642), reflecting the sit-to-stand component present in both tests, while Balance was moderately correlated, though just above the threshold for moderate strength (ρ = −0.430).

### 3.4. Test–Retest Reliability

Table 5 summarizes the test–retest reliability of the SPPB using intraclass correlation coefficients (ICCs). Using a two-way random-effects model, the test–retest reliability of the SPPB was estimated for single scores, based on absolute agreement, in a subsample of 31 participants. The total SPPB score showed good reliability (ICC = 0.893, 95% CI 0.791–0.947), while the Chair subtest also reached good reliability (ICC = 0.882, 95% CI 0.771–0.941). Balance (ICC = 0.688, 95% CI 0.451–0.836) and Gait (ICC = 0.727, 95% CI 0.508–0.858) demonstrated moderate reliability. All estimates were significant (*p* < 0.001).

**Figure 1:**
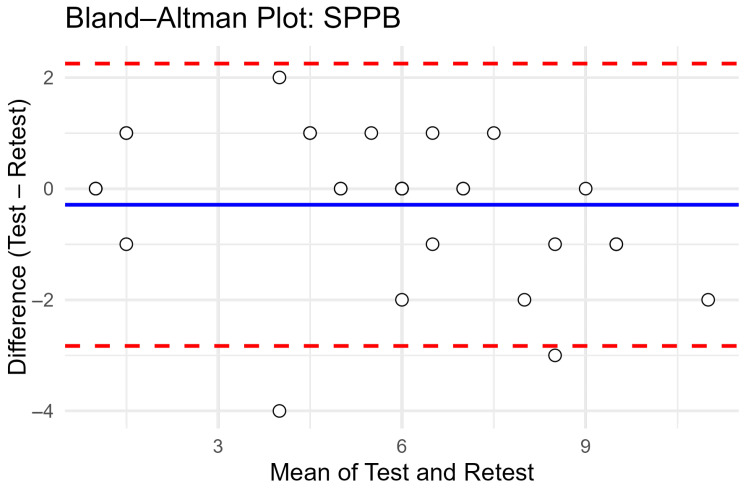
Bland–Altman plot of test–retest agreement for SPPB total score.

The Bland–Altman plot (Figure 1) showed a mean difference close to zero, suggesting no systematic bias between test and retest. The 95% limits of agreement extended from approximately −3.5 to +2.5 points, within which nearly all data points fell. This indicates that while the SPPB is stable at the group level, individual participants may show variations of up to ±3 points across repeated assessments. From a clinical perspective, limits of agreement of approximately ±3 points suggest that individual SPPB scores may vary by up to one functional category across repeated measurements. While this variability is acceptable for group-level comparisons, it should be considered when interpreting small changes in individual patients over short intervals.

### 3.5. Construct Validity: Factorial Structure of the SPPB

Factor analysis was also applied to the SPPB. Sampling adequacy was marginal (overall KMO = 0.69; item MSAs 0.68–0.71), but Bartlett’s test of sphericity was significant (χ^2^(3) = 104.49, *p* < 0.001), indicating sufficient inter-item correlations for analysis. The eigenvalue structure (2.00, 0.52, 0.48) clearly supported the extraction of a single factor. Exploratory factor analysis (principal axis; oblimin rotation) yielded strong standardized loadings for Balance (0.68), Gait (0.73), and Chair (0.72), with communalities ranging from 0.46 to 0.53 and 50% of variance explained.

## 4. Discussion

This study presents the first metric validation of the Short Physical Performance Battery (SPPB) in older adults in Croatia, thereby addressing a relevant methodological gap in national geriatric and sarcopenia-related research. As the SPPB is an integral component of the diagnostic algorithm proposed by the European Working Group on Sarcopenia in Older People (EWGSOP2) [3], the availability of a validated instrument is essential for its appropriate use in clinical practice and research within the Croatian healthcare context.

Previous research using the SARC-F questionnaire has shown that age is the strongest predictor of sarcopenia, with older adults, particularly nursing home residents, being at higher risk compared to community-dwelling individuals [33]. These findings highlight the importance of objective, performance-based assessments, such as the SPPB, for accurately evaluating lower extremity function and identifying older adults at risk of sarcopenia across different living settings.

Complementing these findings, the recent Croatian validation of the SARC-F questionnaire confirmed good reliability (Cronbach’s alpha = 0.76; ICC = 0.86) and significant correlations with SPPB (ρ = −0.50), TUG (ρ = 0.50), and handgrip strength (ρ = −0.42), supporting its convergent validity. This evidence reinforces the value of integrating both self-reported and performance-based measures to comprehensively identify older adults at risk of sarcopenia in community and institutional settings [34].

The SPPB demonstrated acceptable internal consistency, with a Cronbach’s alpha of 0.74. This finding is consistent with previous validation studies conducted in older populations, where Cronbach’s alpha values typically range between 0.70 and 0.78 [14,16,19].

Item–total correlations indicated that all three subtests, balance, gait speed, and chair stand, were meaningfully associated with the total SPPB score. The removal of any individual subtest resulted in a reduction in Cronbach’s alpha, supporting the inclusion of all components and reinforcing the conceptual integrity of the SPPB as a composite measure of lower extremity function.

Convergent validity of the SPPB was supported by a moderate-to-strong negative association with the Timed Up and Go (TUG) test (ρ = −0.684). This finding is consistent with previous studies demonstrating that lower SPPB scores are associated with poorer mobility and longer TUG completion times [35,36]. Additional functional measures or frailty-related indices were not included, which may limit the comprehensiveness of the convergent validity assessment. Future studies could incorporate multidimensional frailty instruments to further explore construct relationships.

Among the individual subtests, gait speed exhibited the strongest correlation with TUG performance, reflecting the central role of walking speed as an indicator of functional capacity and health status in older adults. The chair stand subtest also showed a substantial association with TUG, supporting its relevance as a measure of lower-limb muscle strength and functional mobility. The balance subtest demonstrated a weaker, though still significant, correlation with TUG, suggesting that it captures aspects of postural control that are not fully reflected in time-based mobility tests. This finding highlights the added value of the SPPB as a multidimensional assessment tool.

The test–retest reliability of the total SPPB score was good to excellent, with an intraclass correlation coefficient (ICC) of 0.893 over a 14-day interval. This level of temporal stability is comparable to ICC values reported in previous studies involving both community-dwelling and institutionalized older adults [14,37].

At the subtest level, the chair stand test has generally demonstrated higher test–retest reliability, while balance and gait speed subtests often show more moderate reliability, which may reflect greater intra-individual variability in balance and walking performance due to situational or environmental factors [14,18]. The chair stand test demonstrated the highest reliability, while balance and gait speed showed moderate reliability. These differences are consistent with earlier reports [38,39] and may reflect greater intra-individual variability in balance and walking performance due to situational or environmental factors.

Bland–Altman analysis did not reveal systematic bias between test and retest measurements. However, the observed limits of agreement suggest that individual SPPB scores may vary by up to approximately three points [14]. In our study, the results indicate that within the context of a 0–12 scoring range, such variation may lead to reclassification across adjacent functional categories. Small changes in individual scores should be interpreted with caution, particularly when applied to short-term clinical decision-making.

Exploratory factor analysis supported a single-factor structure, with all three subtests loading strongly on a common latent factor representing lower extremity functional performance. Although the interpretation of our analytic results is limited by the small number of items, the observed unidimensionality is consistent with previous validation studies and with the original conceptualization of the SPPB as a global indicator of lower extremity function and supports the use of the total SPPB score as a summary measure of physical performance [8,16,18].

The mean SPPB score observed in our study was 6.62 (SD = 3.34), suggesting moderate functional impairment in the examined sample, which is comparable to findings reported in older and institutionalized populations in other European settings. For example, a multicenter observational study of institutionalized older adults reported a mean SPPB score of 6.94 ± 3.17, indicating similarly reduced physical performance among nursing home residents. Moreover, systematic reviews of SPPB performance across older samples report mean scores ranging approximately between 7.9 and 11.5 in community-dwelling older adults, further illustrating that lower scores such as those observed in our study, are typical of populations with greater functional limitations [40]. In our study, a substantial proportion of participants were classified as having low or very low functional status, indicating a high burden of functional limitations and a potentially increased risk of sarcopenia. Similar patterns have been reported in other European and older populations, where a large share of older adults presented with low or poor SPPB scores. For example, in a representative European study of very old persons aged 70+ years, 60% of women and nearly 40% of men had low modified SPPB performance, reflecting prevalent functional impairment. Moreover, SPPB cut-off points of ≤8 have been consistently associated with high sensitivity for severe sarcopenia, supporting the clinical relevance of low SPPB scores as an indicator of increased sarcopenia risk. Finally, in studies of older adults with functional limitations after acute health events, SPPB ≤ 8 was present in about 28% and was correlated with physical limitations linked to adverse health outcomes [41].

From both clinical and public health perspectives, the validated Croatian version of the SPPB represents a feasible and standardized instrument for assessing physical performance in older adults. Its short administration time, minimal equipment requirements, and robust metric properties support its use in routine geriatric assessment, sarcopenia screening, rehabilitation monitoring, and population-based research, in line with current international recommendations for functional assessment in older populations [42].

Several limitations should be considered. First, the sample was predominantly female and recruited from selected regions and mixed settings, which may limit generalizability. The high proportion of female participants in the sample may limit the generalizability of the findings to older men. Second, we did not examine responsiveness to change, measurement error parameters (e.g., SEM/MDC), or predictive validity for adverse outcomes, properties highlighted as critical gaps in recent reviews [10]. Third, although exploratory factor analysis supported a unidimensional structure, the SPPB consists of only three subtests, which inherently limits the interpretability of factor analytic findings. With such a small number of indicators, factor analysis provides only a limited examination of latent dimensionality and should be interpreted primarily as supportive rather than definitive evidence of structural validity. The majority of the participants were institutionalized older adults, which may limit representativeness and generalizability to the broader Croatian older population. Institutionalized individuals often present a lower functional status, which may influence score distribution. Future studies should evaluate responsiveness and clinically meaningful change thresholds in Croatian populations and assess predictive validity for falls, hospitalization, institutionalization, and mortality. The cross-sectional design of the study precludes causal inferences.

## 5. Conclusions

This study demonstrates that the Short Physical Performance Battery (SPPB) is a reliable and valid instrument for assessing lower extremity physical performance in older adults in Croatia. The Croatian version of the SPPB showed acceptable internal consistency, good test–retest reliability, and satisfactory convergent and construct validity when compared with established measures of functional mobility and muscle strength. These findings support the use of the SPPB as a standardized tool for evaluating physical function and screening for functional impairment in both community-dwelling and institutionalized older adults. Although the SPPB is recommended within international sarcopenia assessment algorithms as a measure of physical performance severity, the present study did not evaluate sarcopenia diagnosis or prevalence. Owing to its simplicity, feasibility, and robust metric properties, the SPPB can be readily integrated into routine geriatric assessment, clinical practice, and research settings in Croatia. Future studies should examine responsiveness to change and predictive validity for adverse health outcomes in nationally representative samples.

## Figures and Tables

**Table 1:** Basic descriptive parameters of the participants.

	(*n* = 153)
Gender	
Male	32 (20.9%)
Female	121 (79.1%)
Age [years]	
Mean (SD)	81.4 (7.69)
Median [Min, Max]	82.0 [65.0, 97.0]
Height [cm]	
Mean (SD)	165 (8.06)
Median [Min, Max]	164 [148, 185]
Weight [kg]	
Mean (SD)	75.6 (13.0)
Median [Min, Max]	75.0 [48.0, 115]
BMI [kg/m^2^]	
Mean (SD)	27.8 (4.62)
Median [Min, Max]	27.3 [18.2, 46.3]
Dominant Handgrip Strength [kg]	
Mean (SD)	21.4 (6.95)
Median [Min, Max]	21.1 [3.20, 45.2]
Dominant Handgrip Strength—categorized [kg]	
Normal HGS	119 (77.8%)
Weak HGS	34 (22.2%)

**Table 2:** Descriptive parameters of SPPB and TUG tests.

	(*n* = 153)
Balance Test	
Mean (SD)	2.24 (1.55)
Median [Min, Max]	2.00 [0, 4.00]
Gait Test	
Mean (SD)	2.64 (1.07)
Median [Min, Max]	3.00 [1.00, 4.00]
Chair Test	
Mean (SD)	1.67 (1.36)
Median [Min, Max]	1.00 [0, 4.00]
SPPB	
Mean (SD)	6.62 (3.34)
Median [Min, Max]	6.00 [1.00, 12.0]
SPPB—categorized	
Very low	30 (19.6%)
Low	47 (30.7%)
Moderate	35 (22.9%)
High	41 (26.8%)
TUG	
Mean (SD)	16.0 (9.15)
Median [Min, Max]	12.7 [5.60, 52.0]
TUG—categorized	
Normal	42 (27.5%)
Good mobility	80 (52.3%)
Moderate mobility	18 (11.8%)
Reduced mobility	13 (8.5%)

**Table 3:** Internal consistency: Cronbach’s alpha and split-half reliability.

Metric	Value
Alpha if item removed (balance/gait/chair)	0.67/0.65/0.63
Item–total correlations for balance/gait/chair	0.56/0.59/0.58
Cronbach’s alpha (raw/standardized)	0.74/0.75
Average split-half reliability	0.89
Guttman λ4/λ6/λ3/β (minimum)	0.68/0.67/0.75/0.65
Average inter-item correlation (median)	0.50 (0.49)

**Table 4:** Spearman correlations between SPPB, subtests, and TUG.

Measure	TUG (ρ)	TUG (*p*)
Balance	−0.430	<0.001
Gait	−0.752	<0.001
Chair	−0.642	<0.001
SPPB	−0.684	<0.001

**Table 5:** Test–retest reliability: intraclass correlation coefficients.

Measure	ICC (A,1)	95% CI	F (df1, df2)	*p*
SPPB total	0.893	0.791–0.947	17.9 (30, 30.8)	<0.001
Balance	0.688	0.451–0.836	5.60 (30, 30.4)	<0.001
Gait	0.727	0.508–0.858	6.27 (30, 30.8)	<0.001
Chair	0.882	0.771–0.941	15.7 (30, 30.7)	<0.001

## Data Availability

The data presented in this study are available upon request from the corresponding author. The data are not publicly available due to privacy restrictions.

## Abbreviations

The following abbreviations are used in this manuscript:

EFA	Exploratory factor analysis
EWGSOP	European Working Group on Sarcopenia in Older People
HGS	Handgrip strength
ICC	Intraclass correlation coefficient
KMO	Kaiser–Meyer–Olkin measure
SD	Standard deviation
SPPB	Short Physical Performance Battery
STS	Sit-to-stand test
TUG	Timed Up and Go test
WHO	World Health Organization
